# In Later Life

**Published:** 1893-09-30

**Authors:** 


### THE CARE OF THE FEEBLE-MINDED.

### II.—IN LATER LIFE.

IT has been seen that a proportion of children, estimated at rather more than one in a hundred of the whole school population of London, are incapacitated from one cause or another from profiting by the education at present provided for them. Even when due provision shall have been made for their special needs during childhood, the community is face to face with the serious problem of preserving them from danger, and of turning their enfeebled powers to account in later life.

Considerable pains have been taken to trace the after-career of some of these abnormal or partially deficient children from the time when they leave school. The boys are, as a rule, less conspicuous failures than the girls, but probably only because they escape observation. They are usually incurable truants by the time they are fourteen, and rooted in habits of idleness. With eyes so untrained that they cannot distinguish b from d, and hand too helpless to hold a pen, they are yet masters of all the shrewd resources of the London streets. There are plenty of occupations open to them of a kind requiring no skill, but such lads are invariably "out of work." They live from hand to mouth, cultivating a picturesque raggedness, and depending on casual errands and tips. They are expert in guaging the giving powers of the various religious organisations, and have often the most winning manners, with an appearance of being readily influenced for good. They show a marked horror of Homes, which they are aware mean steady occupation, and they drift eventually into the habitual loafer and drunkard. Apparently they are not long lived. It is interesting to learn from the report of Dr. Gover, Inspector H.M. Prisons, that they do not, as a rule, join the criminal classes. He says: "It is alleged that a great deal of crime is due to weak-mindedness. I do not think it is so. I do not suppose that there are 50 weak-minded persons in the whole of the convict prisons (containing about 4,000 persons) at the present moment." Training from early youth in habits of regular industry might do wonders for these boys. With bad habits once thoroughly fixed, there is little to be done with regard to them after they become their own masters.

With the girls it is different. The inquiry instituted by the Charity Organisation Society* into their career after leaving the Poor Law or elementary schools discovers an almost unvarying history of failure and sin. The wasted childhood, supplying no training in self-restraint, and no teaching which can appeal to their limited intelligence, is succeeded by a period of waywardness, impatience of control, and "greedy love of pleasure in any form." Such girls, with whatever caution they are sent out into the world, drift back to the workhouse branded as failures, and their history is but a record of transference from one refuge to another, varied by short intervals of apparent improvement and continual falls. They are a never-ending source of anxiety and bitter disappointment to the self-sacrificing workers in Homes, who, after watching their improvement under kindly discipline, perhaps, for many months, are forced to be witnesses

* The Feeble-Minded. Part II. The Young Person and Adult. (Swan Sonnenschein and Co.)

to their inability to keep the most carefully-chosen situation, or to resist the first temptation which falls in their way. Unlike the boys, they cling to the shelter of the Homes, where, however, save in very exceptional cases, they cannot be permanently retained, and are happiest when fully occupied. They are, almost without exception, unfitted for domestic service, or for looking after themselves.

Now for girls such as these the Charity Organisation Society urges the establishment, by private charity, supplemented by grants from the Poor Law Guardians, of special industrial homes, "where the girls' working powers must be fully utilised, and they must enjoy such a measure of home life that they may be happy and contented, and disposed to remain." Two Homes of this description are at present in existence, one the Aubert Park Home at Highbury, the other at Painswick, and others are being formed. Lady Frederick Cavendish's permanent laundry home for morally deficient fallen girls is on these lines.

A further recommendation is made by the C. O. S. to the effect that "it should be ascertained whether in some of the country workhouses good and suitable provision could not be made for destitute feeble-minded adults, whether men or women, so that they might, as far as possible, be properly classified, supervised, and employed."

The responsibility of the community towards this most unhappy class of persons cannot be too strongly enforced.

